# Constant-time hardware implementation of Modular Inversion with Kaliski’s Algorithm for ECC

**DOI:** 10.1371/journal.pone.0354145

**Published:** 2026-07-28

**Authors:** Khai Nguyen, Tung Nguyen, Hung Nguyen, Linh Tran

**Affiliations:** Integrated Circuits and Systems Laboratory, Ho Chi Minh City University of Technology (HCMUT), VNU-HCM, Ho Chi Minh City, Vietnam; King Fahd University of Petroleum & Minerals, SAUDI ARABIA

## Abstract

This paper introduces a hardware architecture designed for constant-time modular inversion over prime fields, a critical function in Elliptic Curve Cryptography (ECC). We use Kaliski’s Almost Inversion Algorithm to develop an efficient FPGA-based solution. The proposed design guarantees constant-time execution, optimizing modular inverse computations for ECC applications. When synthesized on a Xilinx Kintex-7 FPGA, the accelerator reaches a frequency of 222.9 MHz, occupying 1.7k Slices without using any DSP blocks. This work focuses on improving the speed and resource usage of modular inversion units, specifically for resource-constrained digital environments.

## 1. Introduction

Safeguarding digital infrastructure relies heavily on cryptography, which underlies major security protocols like TLS [1] and SSH [2]. Since its introduction by Koblitz and Miller in 1985 [3], Elliptic Curve Cryptography (ECC) has become a dominant public-key method, favored over RSA for its superior per-bit security [4]. This efficiency has established ECC as a NIST-endorsed standard for information security [5]. Within the ECC framework, modular inversion is a fundamental yet computationally expensive operation, consuming significant time and hardware resources. Consequently, developing high-performance architectures for modular inversion remains a critical research objective.

ECC operations are typically conducted over two primary Galois fields: prime fields GF(*p*) and binary fields GF($2^{n}$) [3,6,7]. While both offer comparable security, GF($2^{n}$) often allows for faster hardware arithmetic due to simplified carry-free operations. However, GF($2^{n}$) lacks flexibility when field parameters change [8]. Modular inversion, essential for these systems, has roots in the Euclidean algorithm for finding the Greatest Common Divisor (GCD) [9]. Stein’s algorithm later improved this by replacing division with shifts and subtractions [10]. The Binary Extended Euclidean Algorithm (bEEA), an extension of Stein’s work [11], is widely used for computing inverses where gcd(a,p)=1. While Fermat’s Little Theorem is another option, bEEA is generally more efficient for large numbers [12], as it avoids complex multi-precision divisions [13]. Kaliski’s method [14] further optimizes bEEA by reducing additions and subtractions, though it requires a final correction step since it computes the inverse for the Montgomery domain.

Previous research by Lee [15], Ghosh [4], and Hossain [12] has explored efficient FPGA architectures for modular inversion, often employing optimized binary inversion algorithms. Other notable FPGA implementations include works by [16–18], the scalable Montgomery-inversion design of Murat et al. [19], and the modular inversion/division architecture of Mrabet et al. [20]. Dong et al. [8] further improved binary algorithms, while Deshpande et al. [13] adopted a Fast Constant-Time GCD approach. More recent efforts have specifically targeted the trade-off between performance and side-channel resistance: Sghaier et al. [21] proposed a modified binary extended Euclidean inverter (MBEEA) resistant to simple power analysis, Li [22] introduced a high-radix (radix-8) modular inversion algorithm to reduce the iteration count, Ji et al. [23] presented constant-time integer and Montgomery inversion accelerators (CT-IMI and CT-CMMI), and constant-time inversion has been integrated into complete ECC datapaths in recent FPGA processors [24,25]. These works confirm that constant-time modular inversion remains an active research target, but they also highlight a persistent tension between throughput, area, and the guarantee of operand-independent execution.

In this paper, a constant-time modular inversion design based on Kaliski’s almost-inverse algorithm is proposed. The contributions of this work are threefold:

- We refine the constant-time Kaliski variant of Savas et al. [26] by folding the post-inversion normalization into a single comparison-and-subtraction correction stage, which removes the data-dependent second halving loop and the extra correction multiplication used in the classical two-phase Montgomery inverse, while preserving a fixed 2*n* + 1-iteration latency.
- We build the 256-bit datapath from two pipelined Brent-Kung parallel-prefix adders and quantify, rather than merely assert, why this network is preferable to the Kogge-Stone alternative for an area- and timing-constrained inverter.
- We provide a compact finite-state controller and a complete Kintex-7 evaluation, comparing area-time (A×T) product and throughput against both classical and recent (2021–2026) designs.

We concentrate on algorithm optimization and improved arithmetic architectures for ECC modular inversion on hardware.

The paper is organized as follows: Section 2 provides the mathematical foundation for ECC and modular inversion. Section 3 details our proposed hardware architecture. Experimental results and comparisons with existing literature are discussed in Section 4, followed by concluding remarks in Section 5.

## 2. Preliminaries

### 2.1. Elliptic curve cryptography

Due to its efficiency and smaller key sizes, ECC has gained widespread adoption. While usable over both ${𝔽}_{p}$ and $GF(2^{m})$, this study focuses on affine coordinates within prime fields ${𝔽}_{p}$. An elliptic curve *E* over ${𝔽}_{p}$ is defined by Equation 1, where *a*,*b* satisfy $16(4a^{3}+27b^{2})\text{≠}0$.


$$\begin{array}{c} E({𝔽}_{p}):=\left\{(x,y)\in {𝔽}_{p}\times {𝔽}_{p}|y^{2}=x^{3}+ax+b\right\}\cup \left\{𝒪\right\} \end{array}$$ (1)

For points *P*_(*x*1,*y*1)_ and *Q*_(*x*2,*y*2)_ in affine coordinates, the point addition *R* = *P* + *Q* is governed by Equation 2 [27]. Similarly, point doubling *R* = 2*P* is described in Equation 3. Both operations heavily rely on modular inversion, making it the primary computational bottleneck.


$$\begin{array}{c} \left\{ \begin{array}{c} x_{3}=\lambda^{2}-x_{1}-x_{2}\\ y_{3}=\lambda (x_{1}-x_{3})-y_{1}\\ \lambda =\frac{y_{2}-y_{1}}{x_{2}-x_{1}} \end{array}\right. \end{array}$$ (2)

$$\begin{array}{c} \left\{ \begin{array}{c} x_{3}=\lambda^{2}-2x_{1}\\ y_{3}=\lambda (x_{1}-x_{3})-y_{1}\\ \lambda =\frac{3x_{1}^{2}+a}{2y_{1}} \end{array}\right. \end{array}$$ (3)

### 2.2. Coordinate system

ECC points can be represented in multiple coordinate systems, with affine and projective being the most common. Affine coordinates use a pair (*x*, *y*), but group operations like addition and doubling require costly modular inversions. Projective coordinates (*X*, *Y*, *Z*) mitigate this by deferring inversion, yet conversion back to affine coordinates still necessitates one modular inversion per scalar multiplication [27]. Even reduced to a single occurrence, this inversion remains important for two reasons. First, it sits on the critical path of every scalar multiplication and is the single most expensive field operation, so its latency directly bounds the achievable throughput. Second, and more importantly for security, this final inversion operates on secret-dependent data; if it is implemented with a variable-time algorithm, its execution time leaks information about the scalar and is exploitable by timing and simple-power analysis. A constant-time, area-efficient inverter is therefore valuable even in a projective-coordinate processor, and is the focus of this work.

### 2.3 Modular Inversion

For a prime field ${𝔽}_{p}$, the modular inverse of a∈[1,p−1] is the unique R∈[1,p−1] satisfying a·R≡1(modp). Because *p* is prime, every non-zero *a* is automatically coprime to *p*, so this inverse always exists. Modular inversion is the most expensive field operation in affine-coordinate ECC, which motivates a dedicated hardware unit.

Algorithm 1 states the constant-time inverter used in this work. It operates on four *n*-bit registers: *u* and *v* hold the running operands (initialized to *p* and *a*), while *r* and *s* accumulate the inverse (initialized to 0 and 1). The loop counter *k* drives the fixed schedule through the termination predicate $\pi_{0}=(k<2n)$, and $\pi_{1}=(v>0)$ with its complement ${\overset{―}{\pi }}_{1}=(v=0)$ separate the inversion phase from the final correction. The remaining predicates $\pi_{2},\ldots ,\pi_{7}$ test the parity of *u* and *v* and the comparisons *u* vs. *v* and *r* vs. *p*; they are mutually exclusive, so exactly one update branch is taken each cycle. Each bracketed line $\left[\pi_{i}\wedge \pi_{j}\right]$ lists the register updates performed when that predicate combination holds, where Δ and Σ denote the two adder outputs.

**Algorithm 1 Constant-Time Algorithm Based on Kaliski’s Method**

**Input:** a∈[1,p−1] and *p* is prime.

**Output:** r∈[1,p−1] where $r=a^{-1}\cdot M$ (mod p)

1: u←p,v←a,r←0 and s←1

2: k←0

3: $\pi_{0}\leftarrow (k<2n),\pi_{1}\leftarrow (v>0),{\overset{―}{\pi }}_{1}\leftarrow (v=0)$

4: **while** $\pi_{0}$ **do**

5:  $\pi_{2}\leftarrow (u$ is even), $\pi_{3}\leftarrow ({\overset{―}{\pi }}_{2}$ and (*v* is even))

$\pi_{4}\leftarrow ({\overset{―}{\pi }}_{2}$ and ${\overset{―}{\pi }}_{3}$ and (*v* is even)),

$\pi_{5}\leftarrow ({\overset{―}{\pi }}_{2}$ and ${\overset{―}{\pi }}_{3}$ and ${\overset{―}{\pi }}_{4}$), $\pi_{6}\leftarrow (r>p)$, $\pi_{7}\leftarrow {\overset{―}{\pi }}_{6}$

6:  [$\pi_{1}$ and $\pi_{2}$]: Δ←r−s,Σ←Δ+s,s←2s,

u←u/2,r←Σ

7:  [$\pi_{1}$ and $\pi_{3}$]: Δ←s−r,Σ←Δ+r,r←2r,

v←v/2,s←Σ

8:  [$\pi_{1}$ and $\pi_{4}$]: $\Delta \leftarrow u-v,\Sigma_{rs}\leftarrow r+s,s\leftarrow 2s,$

u←Δ/2,r←Σ

9:  [$\pi_{1}$ and $\pi_{5}$]: Δ←v−u,Σ←r+s,r←2r,

u←Δ/2,s←Σ

10: [${\overset{―}{\pi }}_{1}$ and $\pi_{6}$]: Δ←r−p,Σ←s+p,r←2Δ,

Σ←Σ/2,s←Σ

11: [${\overset{―}{\pi }}_{1}$ and $\pi_{7}$]: Δ←r−p,Σ←s+p,r←2r,

Σ←Σ/2,s←Σ

12:  k←k+1

13:  $\pi_{0}\leftarrow (k<2n),\pi_{1}\leftarrow (v>0),{\overset{―}{\pi }}_{1}\leftarrow (v=0)$

14: **end while**

15: **if** *r* > 0 **then**

16:  **return** 2·p−r

17: **end if**

18: **return**: p−r

#### 2.3.1. Kaliski’s almost inverse and the proposed modification.

Kaliski’s algorithm [14] computes the modular inverse in two conceptual phases. Let $n=\lceil \log_{2}p\rceil$. The first phase, the *almost Montgomery inverse*, repeatedly applies binary (shift-and-subtract) reductions to the working pair (*u*,*v*), accumulating the result in (*r*,*s*). It terminates with a pair (*r*,*k*) such that


$$\begin{array}{c} r\equiv a^{-1}\;2^{k}\;(\mathrm{mod}\;p),\;n\leq k\leq 2n. \end{array}$$ (4)

The exponent *k* is the number of reduction steps and therefore depends on the magnitude of *a*. A classical second phase then performs k−n (or, depending on the target domain, 2n−k) conditional modular halvings to strip the residual $2^{k}$ factor and return the normalized inverse [26]. Because both the first-phase loop bound and the second-phase halving count are functions of *k*, a direct implementation leaks operand-dependent timing, which is exploitable through simple power or timing analysis.

To remove this leakage, Savas et al. [26] fixed the iteration count to exactly 2*n* and guarded every register update with a set of mutually exclusive predicates $\pi_{i}$, so that the same arithmetic is executed on every cycle regardless of the operand. Our work uses this constant-time variant but modifies its *normalization*. Instead of appending a separate, variable-length second phase, we fold the correction into the fixed-length schedule: once *v* = 0 (predicate ${\overset{―}{\pi }}_{1}$), the two branches $\left[{\overset{―}{\pi }}_{1}\wedge \pi_{6}\right]$ and $\left[{\overset{―}{\pi }}_{1}\wedge \pi_{7}\right]$ of Algorithm 1 keep *r* within the field by conditionally adding the modulus, and a single closing adjustment (lines 15–18) returns either 2p−r or p−r to map the result into [1,p−1]. This eliminates the data-dependent halving loop and the additional correction multiplication of the two-phase formulation, so the unit completes in a constant 2*n* + 1 iterations while remaining fully constant-time. This single-comparison final stage is the optimization referred to in Algorithm 1.

As a consequence of the fixed schedule, Algorithm 1 returns


$$\begin{array}{c} r\equiv a^{-1}\;M\;(\mathrm{mod}\;p),\;M=(2^{n}{)}^{-2}, \end{array}$$ (5)

where the adjuster *M* is a fixed power of two fixed by the iteration count alone and is independent of *a*. Because *M* is a known constant, it is canceled by pre-scaling the input with a single Montgomery multiplication by the precomputed value $r^{2}\mathrm{mod}p$ (with $r=2^{n}$) on the Montgomery multiplier already present in the ECC datapath. The complete data flow is therefore


$$\begin{array}{c} a\overset{\text{ MMM by }r^{2}\;\;}{\to }a\cdot r^{2}\mathrm{mod}p\overset{\text{ Algorithm 1 }\;}{\to }a^{-1}\mathrm{mod}p. \end{array}$$ (6)

This algorithm executes in exactly 2*n* + 1 iterations, offering superior efficiency over the constant-time binary extended Euclidean algorithm (CBEEA), which requires a longer schedule for the same field size.

## 3. Hardware Implementation of Modular Inversion Over GF(*p*)

**Fig 1:**
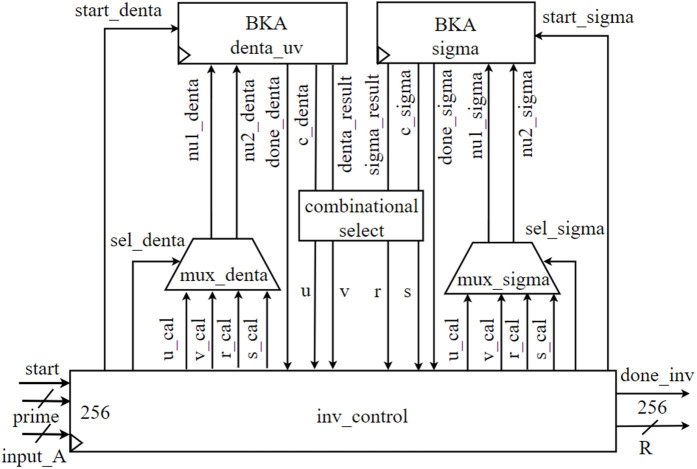
The block diagram of the Modular Inversion Unit.

The overall block diagram of the Modular Inversion Unit is presented in Fig 1. To implement the inversion calculation process, we have two Brent-Kung adders in this design to calculate Δ and Σ in each iteration of the inversion algorithm.

The inversion control unit wires the control signals to the two Brent-Kung adders, analogously to the multiplication control unit. On each cycle it evaluates the predicates of the active algorithm stage (Section 2.3.1) and drives the output control signals that select which register-update branch is executed. In Fig 1, the signals u_cal, v_cal, r_cal and s_cal denote the next-state (temporary) values of the registers *u*, *v*, *r* and *s* produced combinationally by the adders and multiplexers; they are written back into the corresponding registers at the next clock edge. The two adder outputs themselves are labeled Δ and Σ, matching the notation of Algorithm 1.

Lines 15–18 in 1 are added as the final correction stage of the modified Kaliski algorithm. Since Kaliski’s almost inversion method produces an intermediate value rather than a directly normalized modular inverse, the output must be adjusted before it can be used in the ECC datapath. The added correction checks the final value of *r* and maps it back into the valid prime-field range by returning either (2*p* - *r*) or (*p* – *r*). This step ensures that the final result is represented as a positive modular value within the field. In hardware, this correction is simple to implement because it only requires a comparison and subtraction after the fixed iteration loop, avoiding any extra variable-length operation. Therefore, the added lines improve the correctness of the output while preserving the constant-time behavior of the modular inversion unit.

We use the two multiplexers for Δ and Σ to selectively insert the inputs to each Brent-Kung adder in each iteration. The **sel** signals and **start _ delta** with **start _ sigma** is controlled by the **inv _ control** unit.

**Fig 2:**
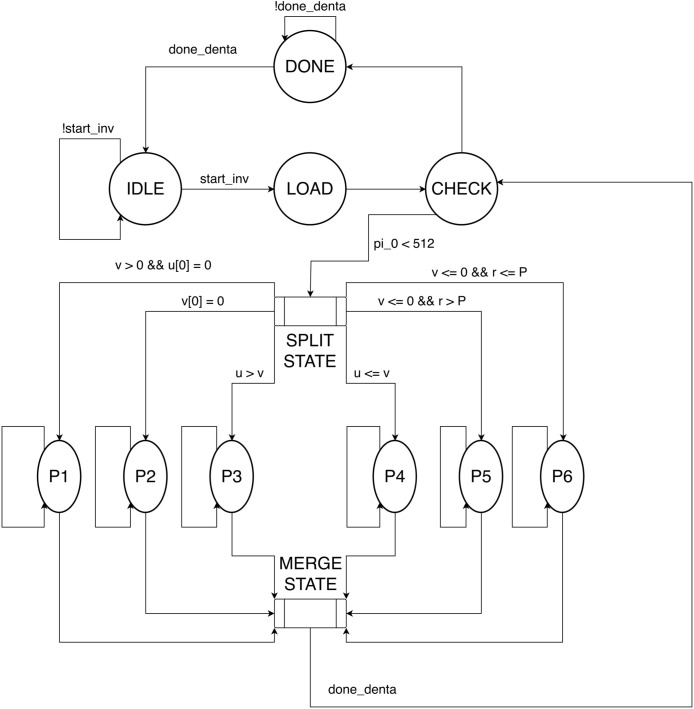
Modular Inversion State diagram.

The finite state diagram designed for the inversion controller unit and each state condition is briefly present as in Fig 2. The state description for each state in the inversion control unit is listed respectively in the Table 1.

**Table 1:** State Description Table for Inversion Control Unit.

State	State Description
IDLE	Wait for the start_inv signal
LOAD	Load inputs (*u* = *p*, *v* = *a*, *r* = 0, *s* = 1)
CHECK	Test the iteration condition $\pi_{0}=(k<2n)$
SPLIT	Select the active branch from predicates $\pi_{1}-\pi_{7}$
P1 → P6	Apply the chosen π-stage register updates
MERGE	Write back *u*,*v*,*r*,*s* and increment *k*
DONE	Apply final correction (2p−r or p−r); assert done

**Fig 3:**
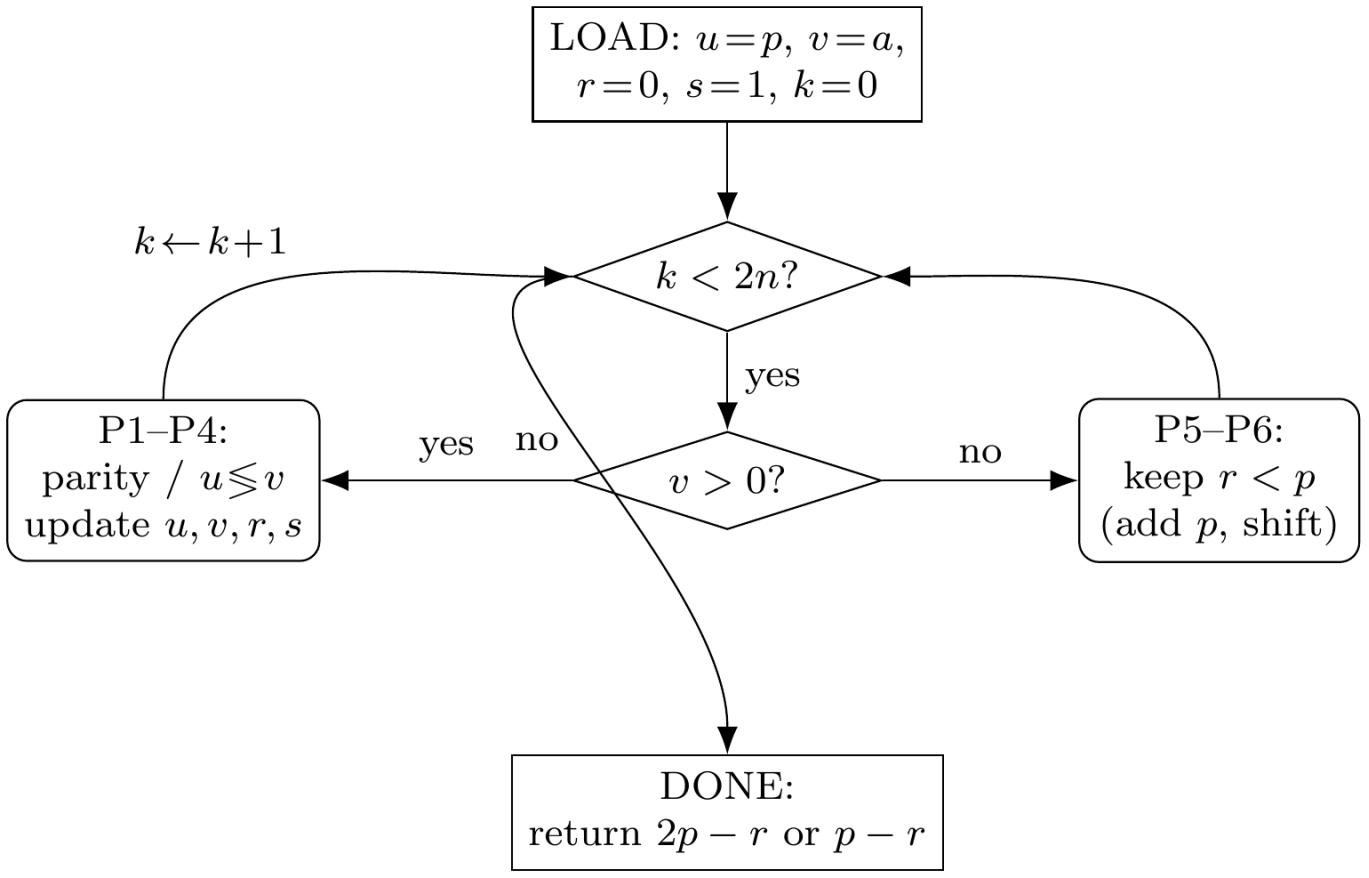
Algorithmic flow chart of the constant-time inversion control.

Fig 2 shows the finite-state machine that controls the proposed Kaliski-based modular inversion architecture, and Fig 3 gives the equivalent algorithmic flow chart of the inversion control. The operation begins in the IDLE state, where the circuit waits for the start_inv signal. Once inversion is triggered, the FSM moves to the LOAD state to initialize the operands and internal registers (u←p, v←a, r←0, s←1), then enters the CHECK state to evaluate the iteration condition $\pi_{0}=(k<2n)$. While the iteration limit has not been reached, control passes to the SPLIT state, which selects one of the processing states P1–P6 according to the mutually exclusive predicates, i.e., the parity of *u* and *v*, the comparison of *u* and *v*, and, once *v* = 0, the comparison of *r* with the modulus *p*. Each processing state corresponds to exactly one bracketed update line of Algorithm 1; the MERGE state then writes the updated registers back, increments *k*, and returns control to CHECK for the next of the 2*n* iterations. When the loop finishes, the FSM enters the DONE state, which applies the final correction (2p−r or p−r) and asserts the done signal to indicate that the modular inverse is ready. This split/merge structure, shown in Fig 2, executes the algorithm as a repeated sequence of register updates suitable for RTL implementation.

### 3.1. Parallel-prefix adder selection

The modular inversion unit relies on repeated 256-bit addition and subtraction, which the FPGA fabric does not provide as a single low-latency primitive at this width. We therefore implement the two operands Δ and Σ with parallel-prefix adders (PPAs), which expose a regular, pipelinable carry tree. The Kogge-Stone adder (KSA) and the Brent-Kung adder (BKA) are the two canonical PPA topologies [28]; they compute the same carries but trade logic depth against the number of prefix operators and routing. For an *n*-bit operand, the KSA reaches the minimum carry-tree depth of $\lceil \log_{2}n\rceil$ but instantiates on the order of $n\log_{2}n-n+1$ prefix cells with a densely interconnected, long-wire network. The BKA uses a deeper carry tree of $2\lceil \log_{2}n\rceil -1$ levels but only about $2n-2-\log_{2}n$ prefix cells arranged in a regular, locally connected layout. Table 2 summarizes these properties for the general *n*-bit case and for the *n* = 256 operands used here.

**Table 2:** Comparison of the two parallel-prefix adder topologies (*n*-bit, and *n* = 256).

Property	Kogge-Stone (KSA)	Brent-Kung (BKA)
Carry-tree depth (levels)	$\log_{2}n$	$2\log_{2}n-1$
at *n* = 256	8	15
Prefix cells (order)	$n\log_{2}n-n+1$	$2n-2-\log_{2}n$
at *n* = 256	≈1793	≈502
Wiring / routing	dense, long wires	sparse, regular
Relative area [29]	higher	lower
Relative power [29]	higher	lower
Pipelineability	limited by routing	high (regular)

For this inverter the choice is governed by area and routability rather than by the latency of a single addition. Each of the 2*n* + 1 iterations issues two 256-bit add/subtract operations, so the adder is replicated and exercised on every cycle; minimizing its cell count and wiring therefore has a larger impact on overall area and achievable clock frequency than shaving one or two prefix levels. The BKA’s roughly 3.5× lower cell count and sparse, regular routing let us split it into a 2-stage pipeline and close timing at 222.9 MHz on the Kintex-7 without DSP blocks, whereas a 256-bit KSA’s routing congestion would inflate area and degrade the attainable frequency. This is consistent with the comparative measurements of [29], which report lower area, delay, and power for the BKA at large word lengths, and with the regular-layout argument of [28]. In short, the BKA performs *n*-bit addition in $O(\log_{2}n)$ time with area on the order of *O*(*n*), making it the better-matched choice for an area- and timing-constrained constant-time inverter.

**Fig 4:**
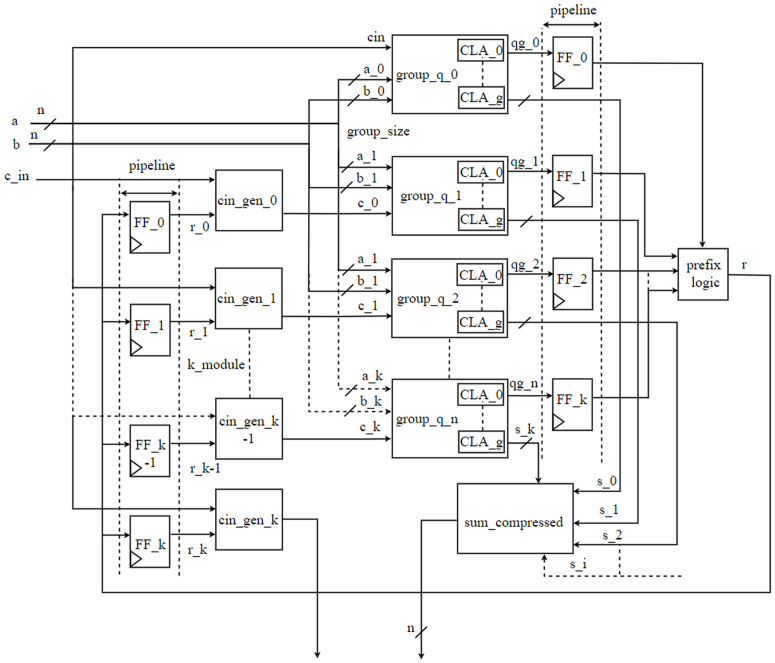
The block diagram of the n-bit Brent-Kung Adder.

The structure of the Brent-Kung adder is designed as a 2-stage pipelined PPA, which provides fast calculations of 256-bit numbers. The BKA provides a solution for our AU core as a faster and more efficient addition with an easy structure for pipelining. Fig 4 presents the structure of a n-bit BKA.

## 4. Result implementation

We simulate and synthesize our design on Xilinx Vivado 23.1, FPGA Platform Kintex-7. The design occupies 1.7k Slices, has no DSP usage, and runs at 222.9 MHz. Our design completed a single modular inversion in 1033 clock cycles. This clock cycle latency means that for a 256-bit prime, we use approximately 2 clocks per Kaliski’s algorithm iteration per bit and do it twice to achieve the final result. Our proposed design’s total latency for one modular inversion is calculated as in Equation 7. The throughput of our design is calculated as in Equation 8.


$$\begin{array}{c} t_{modinv}=(1/F_{max})\times (CCs)\;in\;\mu s \end{array}$$ (7)

$$\begin{array}{c} Throughput=(1/t_{modinv})\times 256\;bits\;in\;Mbps \end{array}$$ (8)

Table 3 compares the proposed architecture against recent related designs with a similar 256-bit structure. Because the surveyed works target different FPGA families and report their cost in different units, a common area measure is needed before they can be compared. Where a design reports look-up tables (LUTs) and flip-flops (FFs) rather than slices, we estimate its slice count using the standard Xilinx slice rule, in which one slice comprises four LUTs and eight FFs; the LUT count dominates this estimate for every surveyed inverter, so we take ⌈LUTs/4⌉ as the equivalent slice count. Designs that report slices directly are used as published, and the proposed design itself uses no DSP blocks.

**Table 3:** The proposed work in comparison with relevant reference designs.

Design	FPGA	Area	Latency	A×T	Throughput
	Platform	Slices	[μs] @ [MHz]	[k-slice$\begin{array}{c} \cdot \mu \end{array}$s]	[Mbps]
Ji et al. CT-CMMI [23]	Virtex-7	0.675k^b^	2.45 @ 116.3	1.65	104.5
Sghaier et al. MBEEA [21]	Virtex-7	2.035k	1.12 @ 276	2.28	228.6
Ji et al. CT-IMI [23]	Virtex-7	1.050k^b^	2.56 @ 115.9	2.69	100
Hossain and Kong^c^ [12]	Virtex-7	1.480k	2.329 @ 146.2	3.45	109.9
Mrabet et al. [20]	Virtex-5	0.592k	7.93 @ 129	4.70	32.28
Murat et al. [19]	Spartan-6	0.275k^b^	22.08 @ 231.9	6.07	11.59
This work	Kintex-7	1.700k	4.63 @ 222.9	7.87	55.3
Deshpande et al.^a^ [13]	UltraScale	0.915k	47.0 @ 190.4	43.01	5.45

^a^ Deshpande et al.’s sequential design; LUT usage not reported.

^b^ Slices estimated from the reported LUT count using the slice rule (one slice ≈ four LUTs).

^c^ The design does not claim constant-time (operand-independent) execution.

This common slice measure enables an Area-Time (A×T) product comparison across different designs, where the area (A) is the total number of slices and the time (T) is the total inversion latency $t_{modinv}$ from Equation 7. Equation 9 shows how the A×T product is calculated; the resulting values are reported in k-slice·μs.


$$\begin{array}{c} A\times T=Number\;of\;Slices\times t_{modinv} \end{array}$$ (9)

**Fig 5:**
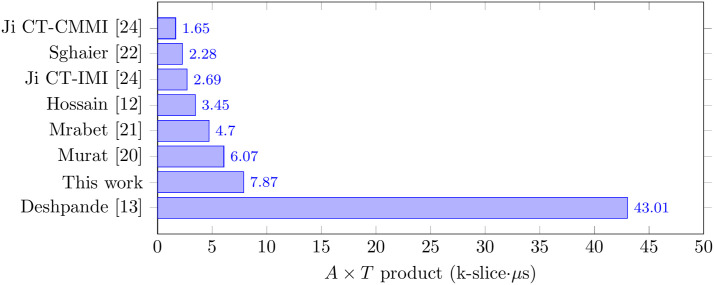
A×T A × T product comparison with related works (Lower is better).

**Fig 6:**
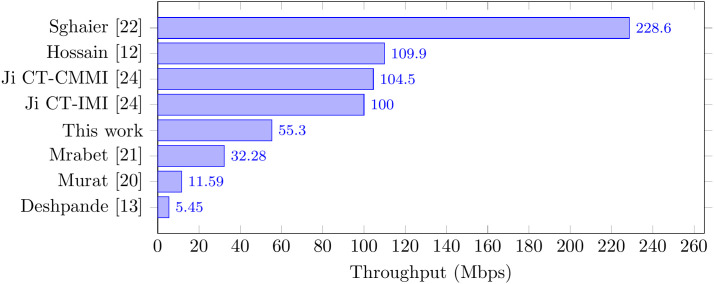
Throughput (Mbps) comparison with related works (Higher is better).

Fig 5 plots the A×T product of every design and Fig 6 the corresponding throughput. The proposed inverter occupies a middle position in both rankings: it improves substantially on the constant-time GCD inverter of Deshpande et al. [13], but the compact recent designs reach a lower A×T and the high-frequency Virtex-7 inverters reach a higher throughput. The proposed design therefore does not claim a best-in-class A×T or throughput; its aim is a constant-time, DSP-free inverter whose cost remains competitive with these works, as discussed below.

The comparison in Table 3 spans several FPGA families and process nodes, from 65 nm Virtex-5–20 nm UltraScale, so the A×T figures should be read as indicative rather than exact: part of the spread reflects fabric and synthesis-tool differences as much as architecture. Within this set the proposed inverter is mid-ranked. The designs that achieve a lower A×T are of two kinds: the recent Virtex-7 accelerators of Sghaier et al. [21] and Ji et al. [23], which reach very low latency on a comparable 28 nm-class fabric, and the small Murat et al. [19] and Mrabet et al. [20] inverters, which trade throughput for a very small footprint. The proposed design is neither the smallest nor the fastest, but it pairs a fully constant-time (operand-independent) schedule with a DSP-free datapath, obtained through the algorithmic normalization of Section 2.3.1 rather than through raw resource minimization.

A per-design comparison clarifies these trade-offs. On a comparable 28 nm-class fabric, Sghaier et al.’s MBEEA [21] is about 4.1× faster and 3.4× more area-time efficient than our design, but uses about 1.2× more slices and follows a different binary extended Euclidean formulation. The two Ji et al. accelerators [23] are likewise constant-time and more compact: CT-IMI is about 1.8× faster with a 2.9× better A×T, and the Montgomery-domain CT-CMMI about 1.9× faster with a 4.8× better A×T, although both figures rest on slice counts we estimated from their reported LUTs. Hossain and Kong’s inverter [12] is about 2.0× faster with a 2.3× better A×T and a slightly smaller area, but it does not provide a constant-time guarantee. Among the smaller designs, the Murat et al. [19] and Mrabet et al. [20] inverters reach a 1.3–1.7× better A×T with much smaller footprints but at 1.7–4.8× higher latency, whereas the Deshpande et al. GCD inverter [13] is about 10× slower and 5.5× less area-time efficient. The proposed design is thus not the most compact or the fastest inverter, but it delivers a constant-time, DSP-free implementation whose area-time cost stays within a small factor of the best published designs while improving substantially on the GCD-based and older binary inverters.

## 5. Conclusion

We have proposed a compact and efficient FPGA implementation of a constant-time modular inversion unit based on Kaliski’s algorithm. By using a Brent-Kung adder and a lightweight control unit, our design achieves 55.3 Mbps throughput. On a Kintex-7 FPGA, it operates at 222.9 MHz using only 1.7k slices and zero DSPs. This demonstrates a viable balance of performance and area for cryptographic applications. Future work will focus on reducing latency to single-cycle operations per bit and integrating this module into larger RISC-V or NoC-based cryptographic systems.

## Supporting information

S1 Data Dataset comparison. (ZIP)
