# Supplementary material for: Constant-time hardware implementation of Modular Inversion with Kaliski’s Algorithm for ECC
Source: PLoS One. 2026 Jul 28;21(7):e0354145. doi: 10.1371/journal.pone.0354145 (PMC13411918; doi:10.1371/journal.pone.0354145)
Supplement: Attachment — Submitted filename: Response_to_Reviewers_PONE-D-25-64818.docx [file pone.0354145.s002.docx]

**Response to Reviewers**

**Manuscript PONE-D-25-64818**

**Title:** Constant-time hardware implementation of Modular Inversion with Kaliski’s Algorithm for ECC

**Journal:** PLOS ONE

Dear Editor and Reviewers,

Thank you very much for the careful and constructive evaluation of our manuscript. We are grateful for the time the reviewers invested and for the specific, actionable suggestions, which have substantially improved the clarity, rigor, and positioning of the paper. We have revised the manuscript thoroughly to address every comment. The main changes are summarized below, and a detailed point-by-point response follows. In the revised manuscript, the locations of each change are indicated by section, equation, figure, and table numbers.

**Summary of the main revisions**

- Added a new Section 2.3.1 that gives the mathematical background of Kaliski’s two-phase inversion and precisely defines our modification, together with Equations (4)–(6).
- Added an explicit, three-part statement of contributions in the Introduction, and expanded the literature with recent (2021–2026) works.
- Added a notation paragraph before Algorithm 1 and a new algorithmic flow chart (Figure 3) to clarify the control logic; clarified the *_cal signal notation of Figure 1.
- Added a new Section 3.1 with a quantitative Kogge-Stone vs. Brent-Kung comparison (Table 2) and a design-specific justification of the adder choice.
- Reworked the Section 4 comparison: the area-time product is recomputed on a common slice basis (k-slice·μs), the design set is focused on recent inverters, and the proposed design's mid-ranked position is reported honestly together with an explicit acknowledgement of the FPGA process-node gap.
- Condensed the modular-inversion paragraph and corrected redundant and minor notational issues throughout.

# **Reviewer #1**

We thank Reviewer #1 for recognizing that the work is technically sound and clearly presented, and for the recommendations to strengthen the novelty positioning, the literature, and the readability of the algorithm and figures.

**Comment 1.1**

*The novelty of the work appears to be incremental, as it is mainly based on existing algorithms and hardware techniques. The contribution should be more clearly positioned with respect to recent literature. (Explain in detail the modification of the author on Kaliski’s algorithm; research more recent documents.)*

**Response:**

We agree that the individual building blocks (Kaliski’s algorithm and parallel-prefix adders) are established, and we have rewritten the paper to state precisely what is new and to position it against recent work.

First, we added a new Section 2.3.1, “Kaliski’s almost inverse and the proposed modification.” It reviews the two conceptual phases of Kaliski’s method: the first phase yields an almost Montgomery inverse r ≡ a⁻¹·2ᵏ (mod p) with n ≤ k ≤ 2n (Eq. 4), and the classical second phase performs k−n operand-dependent modular halvings to normalize it. Because both the loop bound and the halving count depend on k, a direct implementation leaks operand-dependent timing.

Our modification is then defined explicitly: instead of a separate, variable-length second phase, we fold the normalization into the fixed-length 2n+1 schedule. Once v = 0, the two branches guarded by predicates π₆ and π₇ keep r in range, and a single closing comparison-and-subtraction (Algorithm 1, lines 15–18) returns 2p−r or p−r. This removes the data-dependent halving loop and the extra correction multiplication of the two-phase formulation while preserving constant-time behaviour. The resulting adjuster relation and the input pre-scaling that cancels it are given in Equations (5) and (6).

Second, the Introduction now contains an explicit three-part contribution statement (the constant-time normalization refinement; the quantitatively justified Brent-Kung datapath; and a complete Kintex-7 A×T/throughput evaluation against classical and recent designs). We have also expanded the recent literature; please see our response to Comment 1.2.

**Comment 1.2**

*The reference list relies heavily on older works. Including more recent studies (last 4–5 years) would strengthen the paper and improve comparison with current approaches.*

**Response:**

We have added several recent (2021–2026) studies and integrated them into both the discussion and the quantitative comparison. New references include Sghaier et al. (Sensors, 2022, MBEEA), Deshpande et al. (FPL, 2021, constant-time GCD), Li (Computers, 2024, high-radix modular inversion), Ji et al. (ACM TECS, 2026, CT-IMI and CT-CMMI), Wang et al. (Micromachines, 2023), and Hossain et al. (Scientific Reports, 2025). We also added two further FPGA inversion designs that are used in the quantitative comparison, Murat et al. (LightSec, 2011) and Mrabet et al. (ICEMIS, 2017).

The comparison in Table 3 has been rebuilt around the most relevant recent designs: Sghaier et al. (2022), Hossain and Kong, the two Ji et al. (2026) accelerators, the constant-time GCD inverter of Deshpande et al. (2021), and the Murat et al. (2011) and Mrabet et al. (2017) inverters. To make the area comparison consistent, all designs are now placed on a common slice basis and the area-time product is reported in k-slice·μs. This focuses the comparison on current approaches rather than the older Virtex-2/Spartan-3 literature, which has been removed from the table.

**Comment 1.3**

*Some sections, especially the algorithm description and control logic, need clearer explanation for better readability. (Adjust notation on Algorithm 1, redraw Figure 2 with an algorithm flow chart, and explain the notation on Figure 1, e.g. *_cal.)*

**Response:**

We have improved the readability of the algorithm and control-logic description in several ways. A new notation paragraph immediately before Algorithm 1 defines all registers (u, v, r, s), the loop counter k, the adder outputs Δ and Σ, and the meaning of the mutually exclusive predicates π₀–π₇, and explains that each bracketed line lists the updates performed when that predicate combination holds.

To address the request for an algorithm flow chart, we added a new Figure 3 that represents the inversion control flow of Algorithm 1 (LOAD → CHECK → P1–P6 → MERGE/DONE). It complements the finite-state diagram of Figure 2, and the accompanying text now describes both consistently with the state-description table (Table 1).

For Figure 1, we clarified the *_cal notation: the signals u_cal, v_cal, r_cal and s_cal are the combinational next-state (temporary) values of registers u, v, r and s produced by the adders and multiplexers, which are written back to the registers on the next clock edge; the two adder outputs are labelled Δ and Σ, matching Algorithm 1.

**Comment 1.4**

*Figures and diagrams are useful, but their explanation in the text can be improved.*

**Response:**

We have expanded the in-text explanation of every figure: the block diagram (Figure 1) is now described together with its *_cal, Δ and Σ signals; the control flow is described through both the state diagram (Figure 2) and the new flow chart (Figure 3); and the new parallel-prefix adder comparison (Table 2) is discussed in detail in Section 3.1. We believe the figures are now self-consistent with the surrounding text.

**Comment 1.5**

*Minor issues in mathematical notation and formatting should be corrected.*

**Response:**

We have reviewed the notation and formatting throughout. Specific corrections include: removing the redundant coprimality statement in the modular-inversion paragraph (Section 2.3); standardizing the Δ, Σ and π notation between Algorithm 1 and the hardware description; correcting the asymptotic expressions to proper math typesetting, e.g. O(log₂ n); and fixing the grammatical and spelling errors in the adder discussion. We would be glad to correct any further specific items the reviewer identifies.

# **Reviewer #2**

We thank Reviewer #2 for the detailed and rigorous assessment. The comments on the mathematical impact of the modification, the influence of the FPGA technology on the comparison, and the level of detail on the Brent-Kung adders were especially valuable, and we have revised the manuscript accordingly.

**Comment 2.1**

*There does not seem to be any technical innovation. Kaliski’s algorithm with constant time was already referenced by the authors. They did not clearly indicate the impact of their modification or details of it (mathematically). A mathematical review of Kaliski’s algorithm here would have been good. Furthermore, the adder architectures employed are well-known.*

**Response:**

We have addressed this in two ways. The new Section 2.3.1 provides the requested mathematical review of Kaliski’s algorithm (Eqs. 4–6) and states the impact of our modification quantitatively: by folding the normalization into the fixed schedule, we remove the operand-dependent second halving loop and the extra correction multiplication, so the unit completes in a constant 2n+1 iterations while remaining fully constant-time. This is the concrete, measurable effect of the modification.

We also agree that the adder architectures are well known, and we have reframed our claim accordingly. We no longer present the adders as a contribution; instead, the contribution is the constant-time inversion datapath and the algorithmic normalization behind it. The role of the Brent-Kung adder is now justified quantitatively for our specific design rather than asserted (see our response to Comment 2.4).

**Comment 2.2**

*The performance comparison seems highly impacted by the selection of technology: the Kintex-7 FPGA is far superior (28 nm) to Virtex-2 and Spartan-3 older models. It is not established that the gain in speed is due to architectural decisions vs. the choice of FPGA family.*

**Response:**

We fully agree, and the comparison has been substantially reworked to address this. We removed the very old Virtex-2/Spartan-3 designs from the quantitative comparison and now compare mainly against recent inverters on comparable nodes (Virtex-7 28 nm, together with Virtex-5, Spartan-6, and UltraScale parts). We also state explicitly in Section 4 that, where families and process nodes differ, the absolute frequency is influenced by the fabric and is not treated as an architectural result.

To compare area fairly across designs that report their cost in different units, all designs are placed on a common slice basis: where only LUTs and FFs are reported, the slice count is estimated using the Xilinx slice rule (one slice ≈ four LUTs), and the area-time (A×T) product is reported in k-slice·μs. Under this corrected and standardized comparison we do not claim a best-in-class result. The proposed inverter is mid-ranked: several recent designs—Sghaier et al. and the two Ji et al. accelerators on Virtex-7, and the smaller Murat et al. and Mrabet et al. inverters—achieve a lower A×T, while it improves substantially on the older GCD-based inverter of Deshpande et al. We now present the design honestly as a constant-time, DSP-free inverter whose area-time cost stays within a small factor of the best published designs, with the contribution being the algorithmic normalization of Section 2.3.1 rather than a raw efficiency record. A fully controlled comparison would still require re-implementing every reference on an identical device, which is beyond the present scope.

**Comment 2.3**

*The paper is quite short with not a lot of detailed analysis or statistical rigor.*

**Response:**

We have substantially expanded the analytical content of the paper: the mathematical treatment of the inversion algorithm and its modification (Section 2.3.1, Eqs. 4–6); a quantitative parallel-prefix adder analysis with a comparison table (Section 3.1, Table 2); an explicit technology-fairness analysis (Section 4); the algorithmic flow chart (Figure 3); and an updated comparison against recent designs (Table 3). These additions provide the deeper analysis of the datapath and of the speed-up factors that the reviewer requested.

**Comment 2.4**

*Some more rigor in explaining the implementation of the Brent-Kung adders would enrich the delivery. They use it as boxes and rely on references to indicate its superiority to the Kogge-Stone adder. They could shed more light on why they believe this is true for their implementation. (Explain the modification on BKA and KSA with a block diagram of KSA and a comparison table of BKA and KSA.)*

**Response:**

We added a new Section 3.1, “Parallel-prefix adder selection,” with a quantitative comparison table (Table 2) of the Kogge-Stone and Brent-Kung topologies. The table reports the carry-tree depth (⌈log₂ n⌉ vs. 2⌈log₂ n⌉−1), the number of prefix cells (≈ n log₂ n vs. ≈ 2n), the routing characteristics, and the relative area/power, evaluated both generally and for the n = 256 operands used here (8 vs. 15 levels; ≈ 1793 vs. ≈ 502 cells).

Importantly, we now explain why the Brent-Kung adder is the better choice for our specific inverter rather than in general. Because each of the 2n+1 iterations issues two 256-bit add/subtract operations, the adder is replicated and exercised on every cycle, so its cell count and routing dominate area and the achievable clock frequency far more than the latency of a single addition. The Brent-Kung adder’s roughly 3.5× lower cell count and sparse, regular routing allow a 2-stage pipeline that closes timing at 222.9 MHz without DSP blocks, whereas a 256-bit Kogge-Stone network’s routing congestion would inflate area and degrade frequency. The structure of the n-bit Brent-Kung adder is shown in Figure 4.

**Comment 2.5**

*If a projective implementation only requires one inversion, doesn’t that diminish the need for an optimized implementation of this block?*

**Response:**

We have clarified this point in Section 2.2. Even when reduced to a single occurrence per scalar multiplication, the final inversion remains important for two reasons. First, it lies on the critical path and is the single most expensive field operation, so its latency directly bounds the achievable throughput. Second, and more importantly for security, this inversion operates on secret-dependent data; if implemented with a variable-time algorithm, its execution time leaks information about the scalar and is exploitable by timing and simple-power analysis. A constant-time, area-efficient inverter therefore remains valuable even in a projective-coordinate processor, which is precisely the gap this work targets.

**Comment 2.6**

*There is some redundancy in the text (e.g. “provided a and p are coprime,” when it is already established that p is prime). The whole paragraph on modular inversion can almost be omitted.*

**Response:**

We have condensed the modular-inversion paragraph in Section 2.3 and removed the redundant clause: since p is prime, every non-zero a in [1, p−1] is automatically coprime to p, so the inverse always exists, and we now state this once, briefly. We also reviewed the surrounding text for similar redundancy and tightened it.

We hope that the revised manuscript now addresses the reviewers’ concerns regarding novelty, mathematical rigor, the fairness of the comparison, and readability. We thank the editor and the reviewers again for their constructive feedback, which has clearly strengthened the paper, and we remain happy to make any further adjustments.

Sincerely,

**The Authors**
