# Supplementary material for: Constant-time hardware implementation of Modular Inversion with Kaliski’s Algorithm for ECC
Source: PLoS One. 2026 Jul 28;21(7):e0354145. doi: 10.1371/journal.pone.0354145 (PMC13411918; doi:10.1371/journal.pone.0354145)
Supplement: Peer review history

# Peer review: Decision Letter 0

**DOI:** 10.1371/journal.pone.0354145.r001

**Authors:** Muhammad E. S. Elrabaa

17 May 2026

PONE-D-26-05758

Constant-time hardware implementation of Modular Inversion with Kaliski’s Algorithm for ECC

PLOS One

Dear Dr. Tran,

Thank you for submitting your manuscript to PLOS ONE. After careful consideration, we feel that it has merit but does not fully meet PLOS ONE’s publication criteria as it currently stands. Therefore, we invite you to submit a revised version of the manuscript that addresses the points raised during the review process.

Please submit your revised manuscript by Jul 01 2026 11:59PM. If you will need more time than this to complete your revisions, please reply to this message or contact the journal office at plosone@plos.org. When you're ready to submit your revision, log on to https://www.editorialmanager.com/pone/ and select the 'Submissions Needing Revision' folder to locate your manuscript file.

Please include the following items when submitting your revised manuscript:

- A letter that responds to each point raised by the academic editor and reviewer(s). You should upload this letter as a separate file labeled 'Response to Reviewers'.
- A marked-up copy of your manuscript that highlights changes made to the original version. You should upload this as a separate file labeled 'Revised Manuscript with Track Changes'.
- An unmarked version of your revised paper without tracked changes. You should upload this as a separate file labeled 'Manuscript'.

If you would like to make changes to your financial disclosure, please include your updated statement in your cover letter. Guidelines for resubmitting your figure files are available below the reviewer comments at the end of this letter.

If applicable, we recommend that you deposit your laboratory protocols in protocols.io to enhance the reproducibility of your results. Protocols.io assigns your protocol its own identifier (DOI) so that it can be cited independently in the future. For instructions see: https://journals.plos.org/plosone/s/submission-guidelines#loc-laboratory-protocols. Additionally, PLOS ONE offers an option for publishing peer-reviewed Lab Protocol articles, which describe protocols hosted on protocols.io. Read more information on sharing protocols at https://plos.org/protocols?utm_medium=editorial-email&utm_source=authorletters&utm_campaign=protocols.

As the corresponding author, your ORCID iD is verified in the submission system and will appear in the published article. PLOS supports the use of ORCID, and we encourage all coauthors to register for an ORCID iD and use it as well. Please encourage your coauthors to verify their ORCID iD within the submission system before final acceptance, as unverified ORCID iDs will not appear in the published article. *Only*  the individual author can complete the verification step; PLOS staff *cannot*  verify ORCID iDs on behalf of authors.

We look forward to receiving your revised manuscript.

Kind regards,

Muhammad E. S. Elrabaa

Academic Editor

PLOS One

Journal Requirements:

When submitting your revision, we need you to address these additional requirements.

1.Please ensure that your manuscript meets PLOS ONE's style requirements, including those for file naming. The PLOS ONE style templates can be found at

https://journals.plos.org/plosone/s/file?id=wjVg/PLOSOne_formatting_sample_main_body.pdf and

https://journals.plos.org/plosone/s/file?id=ba62/PLOSOne_formatting_sample_title_authors_affiliations.pdf

2. Please note that PLOS One has specific guidelines on code sharing for submissions in which author-generated code underpins the findings in the manuscript. In these cases, we expect all author-generated code to be made available without restrictions upon publication of the work. Please review our guidelines at https://journals.plos.org/plosone/s/materials-and-software-sharing#loc-sharing-code and ensure that your code is shared in a way that follows best practice and facilitates reproducibility and reuse.

3. We note that your Data Availability Statement is currently as follows: “All relevant data are within the manuscript and its Supporting Information files.”

Please confirm at this time whether or not your submission contains all raw data required to replicate the results of your study. Authors must share the “minimal data set” for their submission. PLOS defines the minimal data set to consist of the data required to replicate all study findings reported in the article, as well as related metadata and methods (https://journals.plos.org/plosone/s/data-availability#loc-minimal-data-set-definition).

For example, authors should submit the following data:

- The values behind the means, standard deviations and other measures reported;

- The values used to build graphs;

- The points extracted from images for analysis.

Authors do not need to submit their entire data set if only a portion of the data was used in the reported study.

If your submission does not contain these data, please either upload them as Supporting Information files or deposit them to a stable, public repository and provide us with the relevant URLs, DOIs, or accession numbers. For a list of recommended repositories, please see https://journals.plos.org/plosone/s/recommended-repositories.

If there are ethical or legal restrictions on sharing a de-identified data set, please explain them in detail (e.g., data contain potentially sensitive information, data are owned by a third-party organization, etc.) and who has imposed them (e.g., an ethics committee). Please also provide contact information for a data access committee, ethics committee, or other institutional body to which data requests may be sent. If data are owned by a third party, please indicate how others may request data access.

4. When completing the data availability statement of the submission form, you indicated that you will make your data available on acceptance. We strongly recommend all authors decide on a data sharing plan before acceptance, as the process can be lengthy and hold up publication timelines. Please note that, though access restrictions are acceptable now, your entire data will need to be made freely accessible if your manuscript is accepted for publication. This policy applies to all data except where public deposition would breach compliance with the protocol approved by your research ethics board. If you are unable to adhere to our open data policy, please kindly revise your statement to explain your reasoning and we will seek the editor's input on an exemption. Please be assured that, once you have provided your new statement, the assessment of your exemption will not hold up the peer review process.

5. If the reviewer comments include a recommendation to cite specific previously published works, please review and evaluate these publications to determine whether they are relevant and should be cited. There is no requirement to cite these works unless the editor has indicated otherwise.

Additional Editor Comments:

One of the reviewers raised serious concerns about the novelty of the work as well as its Rigorousness. If you decide to submit a revised version of the manuscript, make sure to address these concerns properly.

[Note: HTML markup is below. Please do not edit.]

Reviewer's Responses to Questions

**Comments to the Author**

1. Is the manuscript technically sound, and do the data support the conclusions?

The manuscript must describe a technically sound piece of scientific research with data that supports the conclusions. Experiments must have been conducted rigorously, with appropriate controls, replication, and sample sizes. The conclusions must be drawn appropriately based on the data presented.

Reviewer #1: Yes

Reviewer #2: No

**********

2. Has the statistical analysis been performed appropriately and rigorously?

Reviewer #1: I Don't Know

Reviewer #2: I Don't Know

**********

3. Have the authors made all data underlying the findings in their manuscript fully available?

The PLOS Data policy requires authors to make all data underlying the findings described in their manuscript fully available without restriction, with rare exception (please refer to the Data Availability Statement in the manuscript PDF file). The data should be provided as part of the manuscript or its supporting information, or deposited to a public repository. For example, in addition to summary statistics, the data points behind means, medians and variance measures should be available. If there are restrictions on publicly sharing data—e.g. participant privacy or use of data from a third party—those must be specified.

Reviewer #1: Yes

Reviewer #2: Yes

**********

4. Is the manuscript presented in an intelligible fashion and written in standard English?

PLOS ONE does not copyedit accepted manuscripts, so the language in submitted articles must be clear, correct, and unambiguous. Any typographical or grammatical errors should be corrected at revision, so please note any specific errors here.

Reviewer #1: Yes

Reviewer #2: Yes

**********

5. Review Comments to the Author

Please use the space provided to explain your answers to the questions above. You may also include additional comments for the author, including concerns about dual publication, research ethics, or publication ethics. (Please upload your review as an attachment if it exceeds 20,000 characters)

Reviewer #1: The manuscript presents a hardware implementation of modular inversion for ECC using Kaliski’s algorithm. The work is technically sound, and the FPGA-based implementation with performance metrics is clearly presented. The comparison with existing approaches is also useful.

However, a few improvements are recommended:

1- The novelty of the work appears to be incremental, as it is mainly based on existing algorithms and hardware techniques. The contribution should be more clearly positioned with respect to recent literature.

2- The reference list relies heavily on older works. Including more recent studies (last 4–5 years) would strengthen the paper and improve comparison with current approaches.

3- Some sections, especially the algorithm description and control logic, need clearer explanation for better readability.

4- Figures and diagrams are useful, but their explanation in the text can be improved.

5- Minor issues in mathematical notation and formatting should be corrected.

Reviewer #2: The manuscript presents an FPGA implementation of a constant-time modular inversion for ECC crypto systems. They emphasize the use of Kaliski's method with a modification, and proceed to give details of the FPGA implementation and measured results.

My main concerns with the manuscript are the following:

1. There does not seem to be any technical innovation. Kaliski's algorithm with constant time was already referenced by the authors. They did not clearly indicate the impact of their modification or details of it (mathematically). I believe a mathematical review of Kaliski's algorithm here would have been good. Furthermore, the adder architectures employed are well-known.

2. The performance comparison seems highly impacted by selection of the technology: Kintex-7 FPGA is far superior (28nm) to Virtex2 and spartan3 older models. The area and timing data is sure to be different. It is not established that the gain in speed is due to architectural decisions vs choice of FPGA family

3. Paper is quite short with not a lot of detailed analysis or statistical rigor

4. Some more rigor in explaining the implementation of the Brent-Kung adders would enrich the delivery. They use it as boxes and rely on references to indicate its superiority to Kogge-Stone adder in all areas. They could shed more light on why they believe this is true for their implementation

5. If a projective implementation only requires one inversion, doesn't that diminish the need for an optimized implementation of this block ?

6. There is some redundancy in the text (e.g. "provided an and p are coprime", when it is already established that p is prime). The whole paragraph on modular inversion can almost be omitted.

I believe a more rigorous analysis and experimentation with critical focus on the data path and the speedup factors would make for a stronger paper. I don't think that it is ready to be published as is.

**********

6. PLOS authors have the option to publish the peer review history of their article (what does this mean?). If published, this will include your full peer review and any attached files.

If you choose “no”, your identity will remain anonymous but your review may still be made public.

**Do you want your identity to be public for this peer review?**  For information about this choice, including consent withdrawal, please see our Privacy Policy.

Reviewer #1: No

Reviewer #2: No

**********

[NOTE: If reviewer comments were submitted as an attachment file, they will be attached to this email and accessible via the submission site. Please log into your account, locate the manuscript record, and check for the action link "View Attachments". If this link does not appear, there are no attachment files.]

To ensure your figures meet our technical requirements, please review our figure guidelines: https://journals.plos.org/plosone/s/figures

You may also use PLOS’s free figure tool, NAAS, to help you prepare publication quality figures: https://journals.plos.org/plosone/s/figures#loc-tools-for-figure-preparation.

NAAS will assess whether your figures meet our technical requirements by comparing each figure against our figure specifications.

# Author comment: Author response to Decision Letter 1

**DOI:** 10.1371/journal.pone.0354145.r002

25 Jun 2026

Dear Editor and Reviewers,

Thank you very much for the careful and constructive evaluation of our manuscript. We are grateful for the time the reviewers invested and for the specific, actionable suggestions, which have substantially improved the clarity, rigor, and positioning of the paper. We have revised the manuscript thoroughly to address every comment. The main changes are summarized below, and a detailed point-by-point response follows. In the revised manuscript, the locations of each change are indicated by section, equation, figure, and table numbers.

Summary of the main revisions

● Added a new Section 2.3.1 that gives the mathematical background of Kaliski’s two-phase inversion and precisely defines our modification, together with Equations (4)–(6).

● Added an explicit, three-part statement of contributions in the Introduction, and expanded the literature with recent (2021–2026) works.

● Added a notation paragraph before Algorithm 1 and a new algorithmic flow chart (Figure 3) to clarify the control logic; clarified the *_cal signal notation of Figure 1.

● Added a new Section 3.1 with a quantitative Kogge-Stone vs. Brent-Kung comparison (Table 2) and a design-specific justification of the adder choice.

● Reworked the Section 4 comparison: the area-time product is recomputed on a common slice basis (k-slice·μs), the design set is focused on recent inverters, and the proposed design's mid-ranked position is reported honestly together with an explicit acknowledgement of the FPGA process-node gap.

● Condensed the modular-inversion paragraph and corrected redundant and minor notational issues throughout.

Reviewer #1

We thank Reviewer #1 for recognizing that the work is technically sound and clearly presented, and for the recommendations to strengthen the novelty positioning, the literature, and the readability of the algorithm and figures.

Comment 1.1

The novelty of the work appears to be incremental, as it is mainly based on existing algorithms and hardware techniques. The contribution should be more clearly positioned with respect to recent literature. (Explain in detail the modification of the author on Kaliski’s algorithm; research more recent documents.)

Response:

We agree that the individual building blocks (Kaliski’s algorithm and parallel-prefix adders) are established, and we have rewritten the paper to state precisely what is new and to position it against recent work.

First, we added a new Section 2.3.1, “Kaliski’s almost inverse and the proposed modification.” It reviews the two conceptual phases of Kaliski’s method: the first phase yields an almost Montgomery inverse r ≡ a⁻¹·2^k^ (mod p) with n ≤ k ≤ 2n (Eq. 4), and the classical second phase performs k−n operand-dependent modular halvings to normalize it. Because both the loop bound and the halving count depend on k, a direct implementation leaks operand-dependent timing.

Our modification is then defined explicitly: instead of a separate, variable-length second phase, we fold the normalization into the fixed-length 2n+1 schedule. Once v = 0, the two branches guarded by predicates π₆ and π₇ keep r in range, and a single closing comparison-and-subtraction (Algorithm 1, lines 15–18) returns 2p−r or p−r. This removes the data-dependent halving loop and the extra correction multiplication of the two-phase formulation while preserving constant-time behaviour. The resulting adjuster relation and the input pre-scaling that cancels it are given in Equations (5) and (6).

Second, the Introduction now contains an explicit three-part contribution statement (the constant-time normalization refinement; the quantitatively justified Brent-Kung datapath; and a complete Kintex-7 A×T/throughput evaluation against classical and recent designs). We have also expanded the recent literature; please see our response to Comment 1.2.

Comment 1.2

The reference list relies heavily on older works. Including more recent studies (last 4–5 years) would strengthen the paper and improve comparison with current approaches.

Response:

We have added several recent (2021–2026) studies and integrated them into both the discussion and the quantitative comparison. New references include Sghaier et al. (Sensors, 2022, MBEEA), Deshpande et al. (FPL, 2021, constant-time GCD), Li (Computers, 2024, high-radix modular inversion), Ji et al. (ACM TECS, 2026, CT-IMI and CT-CMMI), Wang et al. (Micromachines, 2023), and Hossain et al. (Scientific Reports, 2025). We also added two further FPGA inversion designs that are used in the quantitative comparison, Murat et al. (LightSec, 2011) and Mrabet et al. (ICEMIS, 2017).

The comparison in Table 3 has been rebuilt around the most relevant recent designs: Sghaier et al. (2022), Hossain and Kong, the two Ji et al. (2026) accelerators, the constant-time GCD inverter of Deshpande et al. (2021), and the Murat et al. (2011) and Mrabet et al. (2017) inverters. To make the area comparison consistent, all designs are now placed on a common slice basis and the area-time product is reported in k-slice·μs. This focuses the comparison on current approaches rather than the older Virtex-2/Spartan-3 literature, which has been removed from the table.

Comment 1.3

Some sections, especially the algorithm description and control logic, need clearer explanation for better readability. (Adjust notation on Algorithm 1, redraw Figure 2 with an algorithm flow chart, and explain the notation on Figure 1, e.g. *_cal.)

Response:

We have improved the readability of the algorithm and control-logic description in several ways. A new notation paragraph immediately before Algorithm 1 defines all registers (u, v, r, s), the loop counter k, the adder outputs Δ and Σ, and the meaning of the mutually exclusive predicates π₀–π₇, and explains that each bracketed line lists the updates performed when that predicate combination holds.

To address the request for an algorithm flow chart, we added a new Figure 3 that represents the inversion control flow of Algorithm 1 (LOAD → CHECK → P1–P6 → MERGE/DONE). It complements the finite-state diagram of Figure 2, and the accompanying text now describes both consistently with the state-description table (Table 1).

For Figure 1, we clarified the *_cal notation: the signals u_cal, v_cal, r_cal and s_cal are the combinational next-state (temporary) values of registers u, v, r and s produced by the adders and multiplexers, which are written back to the registers on the next clock edge; the two adder outputs are labelled Δ and Σ, matching Algorithm 1.

Comment 1.4

Figures and diagrams are useful, but their explanation in the text can be improved.

Response:

We have expanded the in-text explanation of every figure: the block diagram (Figure 1) is now described together with its *_cal, Δ and Σ signals; the control flow is described through both the state diagram (Figure 2) and the new flow chart (Figure 3); and the new parallel-prefix adder comparison (Table 2) is discussed in detail in Section 3.1. We believe the figures are now self-consistent with the surrounding text.

Comment 1.5

Minor issues in mathematical notation and formatting should be corrected.

Response:

We have reviewed the notation and formatting throughout. Specific corrections include: removing the redundant coprimality statement in the modular-inversion paragraph (Section 2.3); standardizing the Δ, Σ and π notation between Algorithm 1 and the hardware description; correcting the asymptotic expressions to proper math typesetting, e.g. O(log₂ n); and fixing the grammatical and spelling errors in the adder discussion. We would be glad to correct any further specific items the reviewer identifies.

Reviewer #2

We thank Reviewer #2 for the detailed and rigorous assessment. The comments on the mathematical impact of the modification, the influence of the FPGA technology on the comparison, and the level of detail on the Brent-Kung adders were especially valuable, and we have revised the manuscript accordingly.

Comment 2.1

There does not seem to be any technical innovation. Kaliski’s algorithm with constant time was already referenced by the authors. They did not clearly indicate the impact of their modification or details of it (mathematically). A mathematical review of Kaliski’s algorithm here would have been good. Furthermore, the adder architectures employed are well-known.

Response:

We have addressed this in two ways. The new Section 2.3.1 provides the requested mathematical review of Kaliski’s algorithm (Eqs. 4–6) and states the impact of our modification quantitatively: by folding the normalization into the fixed schedule, we remove the operand-dependent second halving loop and the extra correction multiplication, so the unit completes in a constant 2n+1 iterations while remaining fully constant-time. This is the concrete, measurable effect of the modification.

We also agree that the adder architectures are well known, and we have reframed our claim accordingly. We no longer present the adders as a contribution; instead, the contribution is the constant-time inversion datapath and the algorithmic normalization behind it. The role of the Brent-Kung adder is now justified quantitatively for our specific design rather than asserted (see our response to Comment 2.4).

Comment 2.2

The performance comparison seems highly impacted by the selection of technology: the Kintex-7 FPGA is far superior (28 nm) to Virtex-2 and Spartan-3 older models. It is not established that the gain in speed is due to architectural decisions vs. the choice of FPGA family.

Response:

We fully agree, and the comparison has been substantially reworked to address this. We removed the very old Virtex-2/Spartan-3 designs from the quantitative comparison and now compare mainly against recent inverters on comparable nodes (Virtex-7 28 nm, together with Virtex-5, Spartan-6, and UltraScale parts). We also state explicitly in Section 4 that, where families and process nodes differ, the absolute frequency is influenced by the fabric and is not treated as an architectural result.

To compare area fairly across designs that report their cost in different units, all designs are placed on a common slice basis: where only LUTs and FFs are reported, the slice count is estimated using the Xilinx slice rule (one slice ≈ four LUTs), and the area-time (A×T) product is reported in k-slice·μs. Under this corrected and standardized comparison we do not claim a best-in-class result. The proposed inverter is mid-ranked: several recent designs—Sghaier et al. and the two Ji et al. accelerators on Virtex-7, and the smaller Murat et al. and Mrabet et al. inverters—achieve a lower A×T, while it improves substantially on the older GCD-based inverter of Deshpande et al. We now present the design honestly as a constant-time, DSP-free inverter whose area-time cost stays within a small factor of the best published designs, with the contribution being the algorithmic normalization of Section 2.3.1 rather than a raw efficiency record. A fully controlled comparison would still require re-implementing every reference on an identical device, which is beyond the present scope.

Comment 2.3

The paper is quite short with not a lot of detailed analysis or statistical rigor.

Response:

We have substantially expanded the analytical content of the paper: the mathematical treatment of the inversion algorithm and its modification (Section 2.3.1, Eqs. 4–6); a quantitative parallel-prefix adder analysis with a comparison table (Section 3.1, Table 2); an explicit technology-fairness analysis (Section 4); the algorithmic flow chart (Figure 3); and an updated comparison against recent designs (Table 3). These additions provide the deeper analysis of the datapath and of the speed-up factors that the reviewer requested.

Comment 2.4

Some more rigor in explaining the implementation of the Brent-Kung adders would enrich the delivery. They use it as boxes and rely on references to indicate its superiority to the Kogge-Stone adder. They could shed more light on why they believe this is true for their implementation. (Explain the modification on BKA and KSA with a block diagram of KSA and a comparison table of BKA and KSA.)

Response:

We added a new Section 3.1, “Parallel-prefix adder selection,” with a quantitative comparison table (Table 2) of the Kogge-Stone and Brent-Kung topologies. The table reports the carry-tree depth (⌈log₂ n⌉ vs. 2⌈log₂ n⌉−1), the number of prefix cells (≈ n log₂ n vs. ≈ 2n), the routing characteristics, and the relative area/power, evaluated both generally and for the n = 256 operands used here (8 vs. 15 levels; ≈ 1793 vs. ≈ 502 cells).

Importantly, we now explain why the Brent-Kung adder is the better choice for our specific inverter rather than in general. Because each of the 2n+1 iterations issues two 256-bit add/subtract operations, the adder is replicated and exercised on every cycle, so its cell count and routing dominate area and the achievable clock frequency far more than the latency of a single addition. The Brent-Kung adder’s roughly 3.5× lower cell count and sparse, regular routing allow a 2-stage pipeline that closes timing at 222.9 MHz without DSP blocks, whereas a 256-bit Kogge-Stone network’s routing congestion would inflate area and degrade frequency. The structure of the n-bit Brent-Kung adder is shown in Figure 4.

Comment 2.5

If a projective implementation only requires one inversion, doesn’t that diminish the need for an optimized implementation of this block?

Response:

We have clarified this point in Section 2.2. Even when reduced to a single occurrence per scalar multiplication, the final inversion remains important for two reasons. First, it lies on the critical path and is the single most expensive field operation, so its latency directly bounds the achievable throughput. Second, and more importantly for security, this inversion operates on secret-dependent data; if implemented with a variable-time algorithm, its execution time leaks information about the scalar and is exploitable by timing and simple-power analysis. A constant-time, area-efficient inverter therefore remains valuable even in a projective-coordinate processor, which is precisely the gap this work targets.

Comment 2.6

There is some redundancy in the text (e.g. “provided a and p are coprime,” when it is already established that p is prime). The whole paragraph on modular inversion can almost be omitted.

Response:

We have condensed the modular-inversion paragraph in Section 2.3 and removed the redundant clause: since p is prime, every non-zero a in [1, p−1] is automatically coprime to p, so the inverse always exists, and we now state this once, briefly. We also reviewed the surrounding text for similar redundancy and tightened it.

We hope that the revised manuscript now addresses the reviewers’ concerns regarding novelty, mathematical rigor, the fairness of the comparison, and readability. We thank the editor and the reviewers again for their constructive feedback, which has clearly strengthened the paper, and we remain happy to make any further adjustments.

Sincerely,

Attachment Submitted filename: Response_to_Reviewers_PONE-D-25-64818.docx

# Peer review: Decision Letter 1

**DOI:** 10.1371/journal.pone.0354145.r003

**Authors:** Muhammad E. S. Elrabaa

6 Jul 2026

Constant-time hardware implementation of Modular Inversion with Kaliski’s Algorithm for ECC

PONE-D-26-05758R1

Dear Dr. Tran,

We’re pleased to inform you that your manuscript has been judged scientifically suitable for publication and will be formally accepted for publication once it meets all outstanding technical requirements.

Within one week, you’ll receive an e-mail detailing the required amendments. When these have been addressed, you’ll receive a formal acceptance letter and your manuscript will be scheduled for publication.

An invoice will be generated when your article is formally accepted. Please note, if your institution has a publishing partnership with PLOS and your article meets the relevant criteria, all or part of your publication costs will be covered. Please make sure your user information is up-to-date by logging into Editorial Manager at Editorial Manager® and clicking the ‘Update My Information' link at the top of the page. For questions related to billing, please contact billing support.

If your institution or institutions have a press office, please notify them about your upcoming paper to help maximize its impact. If they’ll be preparing press materials, please inform our press team as soon as possible -- no later than 48 hours after receiving the formal acceptance. Your manuscript will remain under strict press embargo until 2 pm Eastern Time on the date of publication. For more information, please contact onepress@plos.org.

Kind regards,

Muhammad E. S. Elrabaa

Academic Editor

PLOS One

Additional Editor Comments (optional):

Please follow any instructions/recommendations provided by the reviewers

Reviewers' comments:

Reviewer's Responses to Questions

**Comments to the Author**

1. If the authors have adequately addressed your comments raised in a previous round of review and you feel that this manuscript is now acceptable for publication, you may indicate that here to bypass the “Comments to the Author” section, enter your conflict of interest statement in the “Confidential to Editor” section, and submit your "Accept" recommendation.

Reviewer #1: All comments have been addressed

Reviewer #2: All comments have been addressed

**********

2. Is the manuscript technically sound, and do the data support the conclusions?

The manuscript must describe a technically sound piece of scientific research with data that supports the conclusions. Experiments must have been conducted rigorously, with appropriate controls, replication, and sample sizes. The conclusions must be drawn appropriately based on the data presented.

Reviewer #1: Yes

Reviewer #2: Yes

**********

3. Has the statistical analysis been performed appropriately and rigorously?

Reviewer #1: N/A

Reviewer #2: N/A

**********

4. Have the authors made all data underlying the findings in their manuscript fully available?

The PLOS Data policy requires authors to make all data underlying the findings described in their manuscript fully available without restriction, with rare exception (please refer to the Data Availability Statement in the manuscript PDF file). The data should be provided as part of the manuscript or its supporting information, or deposited to a public repository. For example, in addition to summary statistics, the data points behind means, medians and variance measures should be available. If there are restrictions on publicly sharing data—e.g. participant privacy or use of data from a third party—those must be specified.

Reviewer #1: Yes

Reviewer #2: Yes

**********

5. Is the manuscript presented in an intelligible fashion and written in standard English?

PLOS ONE does not copyedit accepted manuscripts, so the language in submitted articles must be clear, correct, and unambiguous. Any typographical or grammatical errors should be corrected at revision, so please note any specific errors here.

Reviewer #1: Yes

Reviewer #2: Yes

**********

6. Review Comments to the Author

Please use the space provided to explain your answers to the questions above. You may also include additional comments for the author, including concerns about dual publication, research ethics, or publication ethics. (Please upload your review as an attachment if it exceeds 20,000 characters)

Reviewer #1: The authors have addressed the concerns raised in the previous round of review in a satisfactory manner. The revised manuscript provides a clearer explanation of the proposed modification to Kaliski's algorithm, includes an improved mathematical description, expands the review of recent literature, justifies the selection of the Brent–Kung adder with quantitative analysis, and presents a more balanced and fair comparison with related work. The additional figures and explanations have significantly improved the readability of the manuscript.

The technical contribution is now presented more accurately, and the conclusions are supported by the implementation results and comparative evaluation. The manuscript is technically sound and suitable for publication.

I only recommend a final careful proofreading to correct a few remaining grammatical and stylistic issues, ensure complete consistency of notation throughout the manuscript, and improve the clarity of a few sentences. These are minor editorial issues and do not affect the technical quality of the work.

Overall, I believe the manuscript is suitable for publication in its current revised form.

Reviewer #2: The reviewers have adequately addressed my concerns. The manuscript reads much better to me and gives relevant details to newcomers in the area. The contribution is now stated in more plain English, and should be easy for others to reproduce. I thank the authors for their serious diligence in addressing the concerns head-on, and especially indicating the security implication and information leakage mitigation. I believe the revised manuscript is worthy of publication

**********

7. PLOS authors have the option to publish the peer review history of their article (what does this mean?). If published, this will include your full peer review and any attached files.

If you choose “no”, your identity will remain anonymous but your review may still be made public.

**Do you want your identity to be public for this peer review?**  For information about this choice, including consent withdrawal, please see our Privacy Policy.

Reviewer #1: No

Reviewer #2: **Yes:** Mohammad Waleed Shinwari

**********

# Editor report: Acceptance letter

**DOI:** 10.1371/journal.pone.0354145.r004

**Authors:** Muhammad E. S. Elrabaa

PONE-D-26-05758R1

PLOS One

Dear Dr. Tran,

I'm pleased to inform you that your manuscript has been deemed suitable for publication in PLOS One. Congratulations! Your manuscript is now being handed over to our production team.

At this stage, our production department will prepare your paper for publication. This includes ensuring the following:

* All references, tables, and figures are properly cited

* All relevant supporting information is included in the manuscript submission,

* There are no issues that prevent the paper from being properly typeset

You will receive further instructions from the production team, including instructions on how to review your proof when it is ready. Please keep in mind that we are working through a large volume of accepted articles, so please give us a few days to review your paper and let you know the next and final steps.

Lastly, if your institution or institutions have a press office, please let them know about your upcoming paper now to help maximize its impact. If they'll be preparing press materials, please inform our press team within the next 48 hours. Your manuscript will remain under strict press embargo until 2 pm Eastern Time on the date of publication. For more information, please contact onepress@plos.org.

You will receive an invoice from PLOS for your publication fee after your manuscript has reached the completed accept phase. If you receive an email requesting payment before acceptance or for any other service, this may be a phishing scheme. Learn how to identify phishing emails and protect your accounts at https://explore.plos.org/phishing.

If we can help with anything else, please email us at customercare@plos.org.

Thank you for submitting your work to PLOS ONE and supporting open access.

Kind regards,

PLOS ONE Editorial Office Staff

on behalf of

Dr. Muhammad E. S. Elrabaa

Academic Editor

PLOS One
